# Ridaforolimus (MK-8669) synergizes with Dalotuzumab (MK-0646) in hormone-sensitive breast cancer

**DOI:** 10.1186/s12885-016-2847-3

**Published:** 2016-10-20

**Authors:** Marc A. Becker, Xiaonan Hou, Piyawan Tienchaianada, Brian B. Haines, Sean C. Harrington, S. John Weroha, Sriram Sathyanarayanan, Paul Haluska

**Affiliations:** 1 Department of Oncology, Mayo Clinic, Rochester, MN 55905 USA; 2 Molecular Oncology, Merck Research Laboratories, Boston, MA 02115 USA; 3 Oncology unit, Department of Medicine, Rajavithi Hospital, Bangkok, 10400 Thailand; 4 Division of Medical Oncology, Mayo Clinic College of Medicine, 200 First St. SW, Rochester, MN 55905 USA

**Keywords:** Receptor, IGF type 1, Receptor, Insulin, mTOR inhibitor, Drug resistance, Neoplasm, Breast neoplasms/drug therapy, Aromatase inhibitors/therapeutic use, Disease models, Animal

## Abstract

**Background:**

Mammalian target of rapamycin (mTOR) represents a key downstream intermediate for a myriad of oncogenic receptor tyrosine kinases. In the case of the insulin-like growth factor (IGF) pathway, the mTOR complex (mTORC1) mediates IGF-1 receptor (IGF-1R)-induced estrogen receptor alpha (ERα) phosphorylation/activation and leads to increased proliferation and growth in breast cancer cells. As a result, the prevalence of mTOR inhibitors combined with hormonal therapy has increased in recent years. Conversely, activated mTORC1 provides negative feedback regulation of IGF signaling via insulin receptor substrate (IRS)-1/2 serine phosphorylation and subsequent proteasomal degradation. Thus, the IGF pathway may provide escape (e.g. *de novo* or acquired resistance) from mTORC1 inhibitors. It is therefore plausible that combined inhibition of mTORC1 and IGF-1R for select subsets of ER-positive breast cancer patients presents as a viable therapeutic option.

**Methods:**

Using hormone-sensitive breast cancer cells stably transfected with the aromatase gene (MCF-7/AC-1), works presented herein describe the *in vitro* and *in vivo* antitumor efficacy of the following compounds: dalotuzumab (DALO; “MK-0646”; anti-IGF-1R antibody), ridaforolimus (RIDA; “MK-8669”; mTORC1 small molecule inhibitor) and letrozole (“LET”, aromatase inhibitor).

**Results:**

With the exception of MK-0646, all single agent and combination treatment arms effectively inhibited xenograft tumor growth, albeit to varying degrees. Correlative tissue analyses revealed MK-0646 alone and in combination with LET induced insulin receptor alpha A (InsR-A) isoform upregulation (both mRNA and protein expression), thereby further supporting a triple therapy approach.

**Conclusion:**

These data provide preclinical rationalization towards the combined triple therapy of LET plus MK-0646 plus MK-8669 as an efficacious anti-tumor strategy for ER-positive breast tumors.

**Electronic supplementary material:**

The online version of this article (doi:10.1186/s12885-016-2847-3) contains supplementary material, which is available to authorized users.

## Background

The mammalian target of rapamycin (mTOR) is a master regulator of nutrient sensing (mTORC2) and growth factor signaling (mTORC1) in normal cells and its dysfunction is implicated in the malignant transformation for a variety of cancer types. As activation of mTOR can confer primary resistance to endocrine therapy, a number of prospective clinical trials (Phase II and III) have reported improved survival benefit for hormone receptor-positive breast cancer patients receiving hormone therapy in combination with an mTOR inhibition [1]. However, secondary resistance to mTOR inhibitors remains problematic as suppression of negative feedback inhibition via mTORC1 blockade can induce the IGF type-1 receptor (IGF-1R) and subsequently increase both PI3K and MAPK signaling cascades [2, 3].

The completion of three recent clinical trials, two of which specifically examined MK-0646 and MK-8669, reinforce the added value of targeting IGF-1R in conjunction with mTOR in advanced hormone receptor-positive breast cancer tumors. In a recent report, Di Cosimo and colleagues recently reported that the combination of dalotuzumab (DALO; “MK-0646”; anti-IGF-1R antibody) and ridaforolimus (RIDA; “MK-8669”; mTOR inhibitor) had evidence of antitumor activity in estrogen receptor positive (ER+)/proliferative breast cancer patients that were refractory to multiple chemotherapy regimens. Additional findings from this study suggest that more aggressive ER-positive luminal B subtype tumors exhibited high IGF signaling activation and low RAS pathway activation and that combined MK-8669/MK-0646 combination therapy may be most appropriate for this subset of patients. These data suggest that combination MK-0646 and MK-8669 may be a promising new regimen for ER-positive breast cancer patients whom may have limited benefit from hormonal therapy [4].

To determine the potential anti-tumor benefit of MK-0646 as an effective adjuvant to combination LET and/or MK-8669, xenograft studies were performed in athymic nude mice bearing tumors of hormone-sensitive breast cancer cells stably transfected with the aromatase gene (MCF-7/AC-1). Both *in vitro* and *in vivo* correlative samples were interrogated post-treatment to assess total and/or phosphorylated protein expression (e.g. AKT, S6K1, IGF-1R, MAPK, etc.) post drug administration. In addition, insulin receptor isoform expression was evaluated by qPCR for select treatment subsets. With the exception of MK-0646, all treatments were effective in suppressing tumor growth compared with controls. While MK-8669 further enhanced LET-induced growth inhibition, MK-0646 was less effective than LET alone and LET + MK-0646 was similar to LET alone, likely due to upregulation of InsR-A (confirmed by qPCR and western blot analysis). Insulin signaling through mTOR can be inhibited by the addition of MK-8669, which enhances this activity. Abrogated p70S6K1 and increased Akt phosphorylation confirmed MK-8669 target inhibition. RNAseq analysis revealed MK-0646 alone significantly downregulated IGF/Ins signaling pathway compared to the untreated control tumors and the triple therapy (LET + MK-8669 + MK-0646) significantly impaired the DNA damage repair pathway. While MK-0646 did not significantly enhance LET + MK-8669 tumor growth inihibition, the triple therapy was the most effective therapy *in vivo* to further support its utility in aggressive ER-positive breast cancer tumors.

## Methods

### Cell lines and reagents

Phenol red–free modified IMEM, DMEM, penicillin/streptomycin solution, 0.05 % trypsin-EDTA solution, Dulbecco's PBS, and geneticin (G418) were obtained from Life Technologies. Fetal bovine serum (FBS) and charcoal/dextran–treated FBS were obtained from Hyclone. Androstenedione, tamoxifen (for *in vivo* use), and hydroxypropyl cellulose were obtained from Sigma Chemical Co (St. Louis, MO). Matrigel was purchased from BD Biosciences. Enhanced chemiluminescence [5] kits were purchased from Amersham Biosciences. IGF-1 was purchased from GroPep. Antibodies against p-MAPK, MAPK, AKT, p-AKT, IGF-IRβ and p-IGF-IRβ were purchased from Cell Signaling Technology. An antibody against β-actin was purchased from Sigma-Aldrich. Horseradish peroxidase–conjugated anti-mouse and anti-rabbit secondary antibodies were purchased form Invitrogen. Antibody against insulin Rβ was purchased from Santa Cruz Biotechnology. MCF-7 human breast cancer cells stably transfected with the human aromatase gene (MCF-7/AC-1 cells) were kindly provided by Dr. Angela Brodie and Shiuan Chen (Beckman Research Institute of City of Hope, Duarte, California) as previously reported [6]. Letrozole was purchase from LKT Laboratories, Inc. (Cat# L1878, St Paul, MN, USA). Cells were routinely maintained in DMEM with 10 % fetal bovine serum, 1 % penicillin/streptomycin solution, and 750 ug/mL G418, the culture medium changed twice weekly and origin authenticated by Genetica DNA Laboratories Inc. at the time of study.

### Immunoblotting

For *in vitro* studies, MCF-7/AC-1 cells were cultured in IMEM steroid–reduced medium without phenol red for 24 h prior to treatment initiation with one or more of the following: vehicle control (DMSO), MK-0646 (5, 10 &15 μg/ml), MK-8669 (1, 2 & 3 μmol/L) and Letrozole under serum-free conditions. After 24 h, IGF-1 (10nM) was added to cells for 10 min. Lysates were prepared and analyzed by immunoblot analysis as previously described [7]. Briefly, proteins were extracted from the cell culture lysate or tumor tissues by homogenization in buffer containing 50 mM Tris (pH 7.4), 1 mM EDTA, 150 mM NaCl and proteinase inhibitors (1 μg/ml phenylmethylsulfonyl fluoride, 10 μg/ml aprotinin and 1 μg/ml leupeptin). Homogenates were centrifuged at 2000 g for 15 min at 4 °C. After centrifugation at 10,000 x *g* for 5 min, the supernatants were separated and their protein concentrations were measured. The supernatants were separated by 10 % SDS-PAGE, transferred onto Immuno-Blot polyvinylidene difluoride (PVDF) membrane (catalog no. 162–0177, Bio-Rad), and Western blot analysis was performed. Membranes were blocked with 5 % milk in TBS (10 mM Tris–HCl (pH 8.0) and 150 mM NaCl) plus 0.05 % Tween-20 overnight at 4 °C prior to primary (24 h) and subsequent secondary (1 h) antibody exposure. Proteins of interest were detected using an ECL kit (Amersham, Arlington Heights, IL).

### Clonogenics

Colony-forming assays were used to assess drug effect on cell proliferation. In short, 500 cells obtained after trypsinizing subconfluent cell culture stocks of MCF-7/AC-1 were seeded in 35 mm tissue culture plates (in triplicate) and allowed to adhere overnight. The plated cells were treated with DMSO/diluent (0.1 %) or MK-0646, MK-8669 or MK-0646 plus MK-8669 at the indicated concentrations under serum-free conditions. MCF-7/AC-1 cells were treated with IGF-1 100 nanogram/ml during drug exposure. Following three days of treatment, plates were washed with PBS and media replaced with DMEM (10 % fetal bovine serum, 1 % penicillin/streptomycin solution. The plated cells were allowed to proliferate to form colonies for 7 days. Then plates were washed with PBS, stained with Coomassie blue, and colonies counted on the G:BOX imager system using GeneTools software (Syngene). Results were evaluated graphically using GraphPad PRISM software. Each presented figure is a representative of one of at least three independent experiments, each performed in triplicate. The method of Chou and Talalay was used to determine synergy as described previously [8]. Median effect analysis was done using Calcusyn software (Biosoft). With this method, a combination index (CI) > 1 is deemed antagonistic, a combination index < 1 is synergistic, and combination index = 1 is considered additive. Surface response contours and modeling were carried out in MATLAB as previously described [9].

### Xenograft studies

Female ovariectomized BALB/c athymic nude mice 4–6 weeks of age were obtained from Harlan Laboratories (Indianapolis, IN) and housed in a pathogen-free environment. All animal studies and experiments described herein were carried out and approved according to the guidelines of the Mayo Clinic Institutional Animal Care and Use Committee. Animals were allowed to acclimatize for ≥ 48 h prior to tumor implantation. MCF-7/AC-1 cells were cultured to subconfluency and suspended in Matrigel (10 mg/mL) at a concentration of 2.5 × 10^7^ cells/mL for subcutaneous flank injection (100uL). Mice were randomized to their respective treatment groups using JMP (SAS, Cary, NC) as tumors reached the appropriate size (~250 mm^3^). Tumors were measured weekly with calipers, and volumes calculated according to the following formula: 4/3π x r12 x r2 (r1 < r2), where r1 is the smaller radius. The nonsteroidal aromatase inhibitor Letrozole (10ug/day) and antiestrogen tamoxifen (500 μg/day) were prepared as suspensions (0.3 % hydroxypropyl cellulose) and administered subcutaneously (daily) for 28 days. Dalotuzumab (DALO; “MK-0646”: anti-IGF-1R antibody) was administered weekly via IP injection at a dose of 20 mg/kg for a total of 4 weeks. Ridaforolimus (RIDA; “MK-8669”; mTOR inhibitor) was administered IP at a dose of 1 mg/kg for five continuous days (QDx5) for total 4 weeks. All groups were subcutaneously supplemented with daily androstenedione (100 ug) for the experimental duration as previously described [10]. Mice were monitored daily and weighed once per week. Tumor volume change was calculated when the treatment period was finished, TVC = (ΔT / ΔC) x 100, where ΔT stands for mean tumor volume change of each treatment group and ΔC for mean tumor volume change of control group. Tumor volume and body weight data were analyzed by one-way ANOVA test (GraphPad). At the time of euthanasia, tumors were rapidly excised, weighed and preserved for downstream correlative analyses as snap-frozen and in RNA Later (Invitrogen Life Technologies, Carlsbad, CA, USA).

### Quantitative PCR

Total RNA was isolated using Trizol reagents (Cat#15596-026, Invitrogen, Carlsbad, CA). RNA was reverse transcribed using ABI High capacity RNA to cDNA kit (Applied Biosystem, Carlsbad, CA) as per the manufacturer’s instructions. Sample RNA quality was assessed within each group post reverse-transcriptionally through ribosomal protein 19 (RPL19) expressions. Normalization ratios for each sample were calculated (Nsample = RPL19median/RPL19sample) and compared to the group mean. Samples with RPL19 normalization ratios greater than +3.5 standard deviations (SD) (approximately 99.9 % confidence interval) from the mean were excluded from further analysis. The standard replicates for each qPCR assay were examined for amplification efficiencies between 95–105 % and all standards and sample replicate data were analyzed for product specific melt cures. Sample or standard replicates which did not conform to these parameters were removed from the analysis. Gene transcript copy numbers from each conforming replicate were normalized to RNA input and RPL19 gene expression before being reported as copies of target gene per μg RNA as previously described [11].

### RNA-seq

MCF-7/AC-1 xenograft tumor RNA integrity was confirmed using the Agilent 2100 Bioanalyser (Agilent Technologies, USA) and next-generation sequencing was performed at Merck Co using the Illumina Hiseq 2000 platform (paired-end sequencing, 100 base pairs). RNA-seq data was analyzed with Partek Flow as previously described. Briefly, reads were mapped to the human genome (Ensembl GRCh37) and quantified using the STAR aligner, aligned reads to genes using Partek E/M and differentially expressed genes determined using gene-specific analysis (GSA).

## Results

### IGF-1R inhibition (MK-0646) does not enhance hormonal therapy (letrozole or tamoxifen) in MCF-7/AC-1 xenografts

**Fig. 1:**
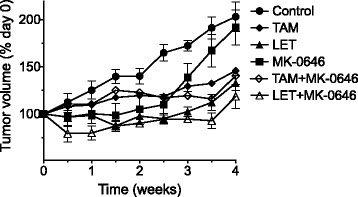
IGF-1R inhibition (MK-0646) does not enhance hormonal therapy (letrozole or tamoxifen) in MCF-7/AC-1 xenografts. Ovariectomized female nu/nu mice between the ages of 7–8 weeks old were inoculated with MCF-7/AC-1 tumor cells in each flank and immediately supplemented with androstenedione (100 μg/day). Once bilateral flank tumors (both *left* and *right*) reached the appropriate size (250–300 mm^3^), mice were randomized (*n* ≥ 8 mice/cohort) and the appropriate treatments initiated (control, MK-0646, letrozole (LET), LET + MK-0646, tamoxifen (TAM), TAM + MK-0646) for a total of 28 days. Tumor volumes were measured twice weekly and depicted as percent change relative to day 0. Error bars represent SEM and results are representative of three independent experiments

Based on our previous investigations demonstrating enhanced activity of hormonal therapy in combination with IGF inhibition, the *in vivo* anti-tumor potential of MK-0646 plus letrozole (LET) or tamoxifen (TAM) was determined [10] (Fig. 1). MCF-7/AC-1 xenografts were established and as tumors reached the appropriate size (250 mm^3^) mice were randomized to one of the following treatment cohorts (*n* ≥ 4 mice/cohort) for a duration of 28 days: Control (vehicle alone), TAM, LET, MK-0646, TAM + MK-0646, LET + MK-0646. Hormonal therapy alone significantly inhibited tumor growth as compared to control and LET was superior to TAM in terms of percent inhibition (34.6 vs. 28.6 %) and statistical significance (*P* < 0.001 vs. *P* < 0.05). While MK-0646 alone did not significantly inhibit tumor growth, the combination LET + MK-0646 was more effective than combination TAM + MK-0646 (41.7 vs. 30.9 %) and resulted in the greatest degree of tumor growth inhibition. Of note, all treatments were well tolerated as bodyweight was maintained throughout study duration (data not shown).

### MK-0646 increases Insulin Receptor A Isoform expression in MCF-7/AC-1 xenografts

**Fig. 2:**
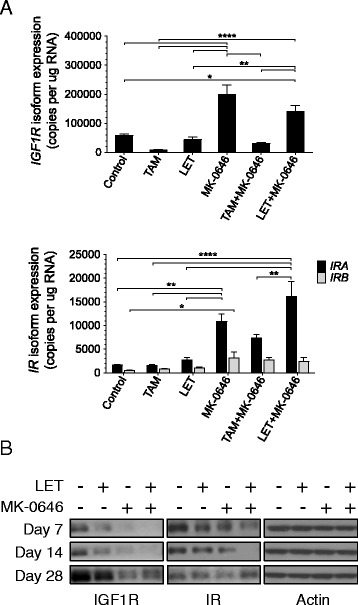
MK-0646 increases Insulin Receptor A Isoform expression in MCF-7/AC-1 xenografts. MCF-7/AC-1 xenograft tumors were harvested and immediately flash frozen following 28 days of treatment (Fig. 1). RNA and protein were extracted for Insulin Receptor and IGF-1R quantification by qPCR (**a**) and western blot analysis (**b**) as described in the methods. **a** Absolute *IGF1R* (*Top*) and *IRA/B* isoform (*Bottom*) copy number normalized to *RPL19* housekeeper. **b** Protein from pooled group replicates isolated at indicated time points were subject to western blotting with indicated antibodies, as described in Materials and Methods section. Error bars represent SEM. *, *P* < 0.05; **, *P* < 0.01; ***, *P* <0.001; **** *P* < 0.0001

In contrast to a small molecule inhibitor targeting IGF-1R and insulin receptor (IR), MK-0646 failed to improve anti-tumor response to hormonal therapy in ER-positive breast cancer xenografts (Fig. 1). To delineate potential compensatory mechanisms, post-treatment tumors were assessed for IGF1R and IR isoform gene expression (Fig. 2). MK-0646 significantly increased both *IGF1R* and *IR* isoform gene expression, *IRB* to a lesser extent as compared to *IRA* (Fig. 2a). While the addition of hormonal therapy had little impact, overall *IRA* expression was highest in LET + MK-0646 tumors (Fig. 2a). MK-0646 induces receptor internalization, leading to subsequent degradation and a reduction in IGF-1R protein levels. Treatments with LET and/or MK-0646, as in Fig. 1, were repeated, but were also terminated at days 7 and 14 days to investigate potential temporal modulations in receptor expression (Fig. 2b). Regardless of time point, IGF-1R expression was markedly reduced in all MK-0646 treatment cohorts. While MK-0646 alone decreased IR expression, the combination LET + MK-0646, following an initial decline at Days 7 and 14, resulted in the highest level of IR expression at Day 28. Statistical analyses for *IGF1R*, *IRA* and *IRB* are included (Additional file 1: Table S1; Additional file 2: Table S2; Additional file 3: Table S3).

### Persistent mTOR signaling in response to IGF-1R inhibition is overcome by MK-8669

**Fig. 3:**
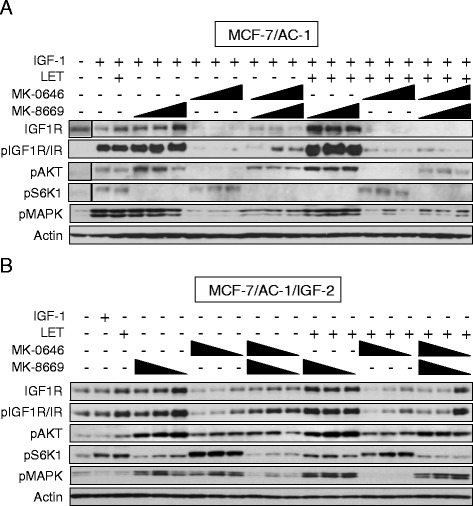
Persistent mTOR signaling in response to IGF-1R inhibition is overcome by MK-8669. Western Blot analysis of MCF-7/AC-1 (**a**) and MCF-7/AC-1/IGF-2 (**b**) cells treated with one or more of the following agents for 24 h at the indicated dose(s): LET (1uM), MK-0646 (1.25, 2.5, 5 ng/uL) and/or MK-8669 (100, 500, 1000 nM). IGF-1 was added for 10 min prior to harvest for the indicated samples

Based on the aforementioned finding where MK-0646 upregulated *IRA* levels, potential compensatory mechanisms (e.g. IR signaling via mTOR) resulting from IGF-1R/ER-targeted inhibition were examined (Fig. 3). Prior to IGF-induced IGF-1R activation, serum-starved MCF-7/AC-1 cells were incubated for 24 h with LET and/or increasing concentrations of MK-0646 and/or MK-8669 (Fig. 3a). As expected, IGF-1 ligand resulted in robust IGF-1R phosphorylation in MCF-7/AC-1 cells. MK-0646, alone or in combination with LET, prevented IGF-induced IGF-1R, Akt and MAPK phosphorylation but induced S6K1 activation. Inhibition of upstream mTOR via MK-8669 effectively blocked S6K1 phosphorylation and in conjunction with previous findings, increased AKT phosphorylation likely via negative feedback loops secondary to mTOR inhibition. More importantly, combination LET + MK-0646 + MK-8669 resulted in the most profound inhibition of IGF-1R/IR, AKT, MAPK and S6K1.

In an effort to simulate IR-A overexpression and its subsequent compensatory activation in response to MK-0646, IGF-2 (known to activate IR-A) was overexpressed (MCF-7/AC-1/IGF-2) and the aforementioned drug combinations were tested (Fig. 3b). As a negative control, IGF-1 did not induce IGF-1R activation. The effects of single agent MK-0646 and MK-8669 were similar to the parental cells in terms of phosphorylated IGF-1R/IR, Akt and S6K1. Although both inhibitors increased MAPK phosphorylation, combination MK-0646 + MK-8669 effectively restored basal levels of both MAPK and IGF-1R/IR phosphorylation. In contrast to MK-8669, where the addition of LET had no effect, MAPK quiescence was achieved following LET + MK0646 treatment. With the exception of MAPK, the triple combination LET + MK-0646 + MK-8669 remained highly effective towards downstream signaling blockade. These data indicate IR-A-induced signaling as a potential escape pathway to IGF/mTOR/ER inhibition. Additional efforts are currently underway to better understand the dynamic signaling interactions in parental vs. MCF-7/AC-1/IGF-2 cells.

### MK-0646 and MK-8669 synergistically inhibit clonogenesis

**Fig. 4:**
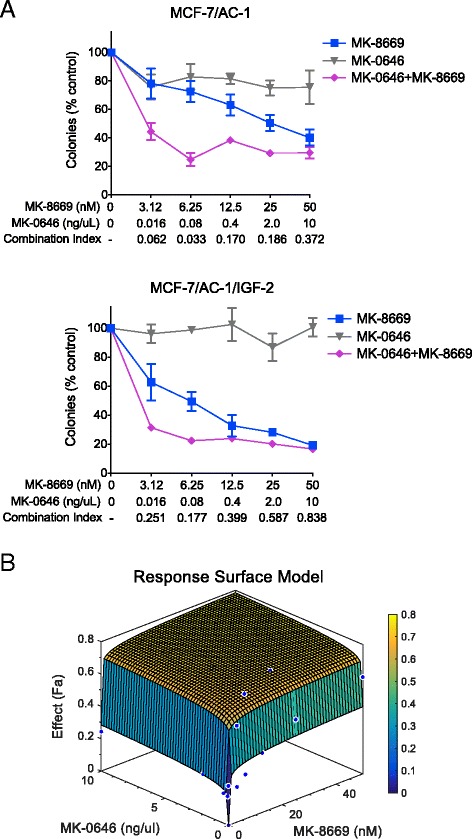
MK-0646 and MK-8669 synergistically inhibit clonogenesis**. a** MCF-7/AC-1 (*Top*) and MCF-7/AC-1/IGF-2 (*Bottom*) cells were exposed to increasing concentrations of MK-8669 (closed squares), MK-0646 (closed triangles) or the combination MK-8669 + MK-0646 (closed diamonds) and assessed for colony outgrowth (normalized to control). Synergy is indicated by combination index (CI) values, where < 1 is synergistic. Error bars represent SEM and results representative of three independent experiments. **b** Surface response modeling was employed to demonstrate the multiplicative benefit of adding MK-8669 to existing MK-0646 or vice versa. MCF-7/AC-1 cells were treated with MK-0646 (x-axis), MK-8669 (y-axis) or the combination (z-axis) at varying doses. Percent inhibition is depicted as the fraction affected (Fa)

Based on the above findings, the synergistic potential of combined mTOR/IGF-1R inhibition was examined (Fig. 4). Specifically, colony formation in response to increasing doses of MK-0646, MK-8669 or the combination MK-0646 + MK-8669 was determined in MCF-7/AC-1 and MCF-7/AC-1/IGF-2 cells (Fig. 4a). MK-0646 alone elicited little (MCF-7/AC-1) to no effect (MCF-7/AC-1/IGF-2). Conversely, MCF-7/AC-1/IGF-2 cells were more sensitive to MK-8669 (IC_50_ = 5.9nM vs. 27.2nM). While the combination resulted in synergy across cell lines, the magnitude of synergy varied as combination index (CI) values were superior in MCF-7/AC-1 vs. MCF-7/AC-1/IGF-2 cells (e.g. 0.062 vs. 0.251). Surface response modeling was employed to illustrate the synergistic potential of MK-0646 + MK-8669 in MCF-7/AC-1 cells (Fig. 4b). These data support multiplicative benefit of adding MK-0646 to existing MK-8669 in ER-positive breast cancer cells and further implicate the IGF-2/IR-A signaling axis as a potential resistance pathway. The remaining works presented herein center upon elucidating the *in vivo* impact and correlative molecular repercussions of said targeted therapies in parental MCF-7/AC-1 xenografts.

### *In vivo* activity of LET +/− MK-8669 +/− MK-0646 in MCF-7/AC-1 xenografts

**Fig. 5:**
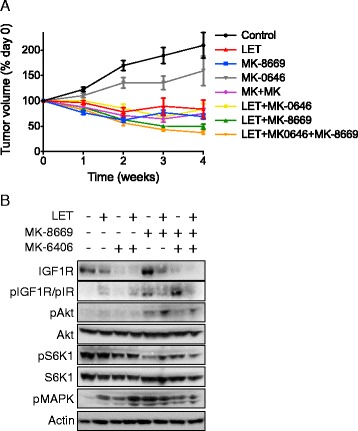
*In vivo* activity of LET +/− MK-8669 +/− MK-0646 in MCF-7/AC-1 xenografts. **a** As previously described, ovariectomized female nu/nu mice between the ages of 7–8 weeks old were inoculated with MCF-7/AC-1 tumor cells in each flank and immediately supplemented with androstenedione (100 μg/day). Once bilateral flank tumors (both *left* and *right*) reached the appropriate size (250–300 mm^3^), mice were randomized (*n* ≥ 9 mice/cohort) and the appropriate treatments initiated (Control, LET, MK-8669, MK-0646, MK-8669 + MK-0646, LET + MK-0646, LET + MK-8669 or LET + MK-0646 + MK-8669) for a total of 28 days. Tumor volumes were measured weekly and depicted as percent change relative to day 0. Error bars represent SEM and results are representative of two independent experiments. **b** Tumors from treatment groups were collected 28 days post treatment initiation, immediately snap frozen and lysates pooled (*n* ≥ 3 samples/treatment cohort) for Western Blot analysis

To determine if the described *in vitro* MK-0646 + MK-8669 synergy translated *in vivo*, established MCF-7/AC-1 xenografts were allowed to reach the appropriate size (250 mm^3^) and mice randomized to one of the following 28-day treatment cohorts (*n* ≥ 7 mice/cohort): Control, LET, MK-8669, MK-0646, MK-8669 + MK-0646, LET + MK-0646, LET + MK-8669 or LET + MK-0646 + MK-8669. While the impact of MK-0646 on tumor growth remained negligible, single agent MK-8669 and LET led to significant tumor regression compared to untreated control (Fig. 5a). Although not significant compared to LET + MK-8669, the triple combination LET + MK-8669 + MK-0646 led to the largest numerical regression compared to untreated control tumors (62.7 % reduction). Statistical analyses comparing treatment cohorts are included (Additional file 4: Table S4). In addition, all agents (both alone and in combination) were well tolerated, as bodyweight did not significantly differ across cohorts throughout treatment duration (data not shown).

To investigate the biochemical effect(s) of the aforementioned therapies, post treatment tumors were harvested at treatment end (Day 28) and subjected to western blotting (Fig. 5b). As demonstrated *in vitro*, single agent efficacy was confirmed as MK-0646 downregulated IGF-1R and MK-8669 resulted in both decreased S6K1 phosphorylation and, albeit to a lesser extent, increased Akt phosphorylation. In addition, MK-8669 led to increased IGF-1R/IR phosphorylation and MK-8669 + MK-0646 further enhanced levels, the result(s) of which may be attributable to IR compensation. Compared to LET + MK-8669, the triple combination LET + MK-8669 + MK-0646 was most effective in terms of decreased total IGF-1R and phosphorylated IGF-1R/IR, Akt, S6K1 and MAPK. These data support co-targeting IGF-1R and mTOR in ER-responsive breast cancer.

### RNA-seq analysis reveals both overlapping and distinct gene regulation in MCF-7/AC-1 xenografts

**Fig. 6:**
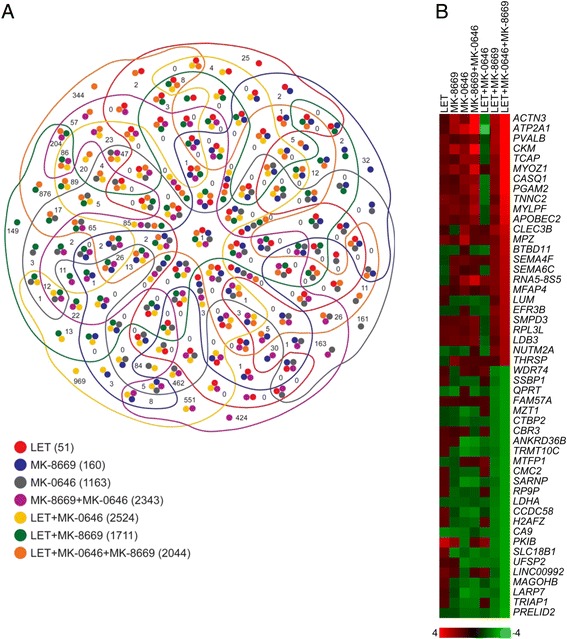
RNAseq analysis reveals both overlapping and non-overlapping gene expression patterns in MCF-7/AC-1 xenografts. **a** Venn Diagram of the overlapping and unique number of genes according to treatment cohort(s). **b** Heatmap depicting the most significantly up (*red*) and down (*green*) regulated genes in LET + MK-8669 + MK-0646-treated tumors compared to the remaining treatment cohorts. Color bar indicates log-fold expression change compared to control tumors

To better understand the transcriptional impact and potential changes in the molecular landscape resulting from targeted IGF-1R, mTOR and ER inhibition*,* an unbiased, comprehensive analysis of post-treatment tumors by RNA-seq was performed (Fig. 6). A complete list of all treatment-induced gene expression changes according to directional magnitude are included (Additional file 5: Table S5). As opposed to *in vivo* tumor repression, where single agent LET and MK-8669 were significantly better than MK-0646, ER (51 genes) and mTOR (160 genes) inhibition altered few genes compared to IGF blockade (1,163 genes). However, when combined with one another, major increases were observed (e.g. 1,711 genes in LET + MK-8669). In an effort to better understand gene expression changes across cohorts, treatments were normalized to controls and presented as a multi-set Venn diagram (Fig. 6a). Depending on the contrast, overlapping (e.g. LET + MK-8669 vs. LET + MK-8669 + MK-0646) or distinct (e.g. LET + MK-8669 vs. LET + MK-8669 + MK-0646) gene expression patterns were observed. A heatmap depicting the most significantly regulated genes in the LET + MK-8669 + MK-0646 cohort illustrate the co-regulatory nature (both direction and magnitude) with LET + MK-8669 (Fig. 6b). Upon close examination, the majority of downregulated genes have been linked a more aggressive breast tumor phenotype. However, numerous upregulated genes have associated with breast tumor progression and metastasis, suggesting possible compensatory transcriptional alterations to overcome treatment-induced anti-tumor effects.

Functional analysis was performed in IPA (Ingenuity Pathway Analysis) was performed to assess global transcription (Additional file 6: Table S6). Although MK-0646 was the only treatment that failed to inhibit *in vivo* tumor growth, antibody efficacy was first confirmed as ‘IGF-1 Signaling’ was predicted as a significantly inhibited pathway in the MK-0646 cohort. Using *p*-value and z-score, the ‘The Role of BRCA1 in DNA Damage Response’ was predicted as the top canonical pathway across treatments (Additional file 7: Figure S1). A heatmap of pathway-specific genes within each treatment confirmed that DNA damage response was most impaired in the LET + MK-8669 + MK-0646 cohort (Additional file 8: Figure S2). Therefore, combined ER/mTOR/IGF targeting may greatly sensitize tumors to DNA-damaging agents (e.g. standard chemotherapy) and as a result, efforts to test the triple therapy with standard of care chemotherapies are being explored.

## Discussion

A plethora of preclinical data supports targeted inhibition of the IGF pathway across numerous cancer types [12]. Unfortunately, a general lack of activity is a common stigmata of IGF-1R inhibitors in breast cancer [13]. To highlight this point, a randomized, controlled, double-blind, phase 2 trial examining the potential benefit of adding the IGF-1R inhibitor ganitumab (AMG 479) to hormonal therapy in patients with hormone-receptor positive breast cancer reported negative results [14]. The failure of ganitumab and other IGF-targeting agents has been widely attributed to poor patient selection (e.g. lack of predictive biomarkers) and/or unforeseen compensatory signaling networks (e.g. HER, IR, IGFBPs) [15–18]. As a result, additional studies to identify, and potentially target, the key factors mediating anti-IGF-1R resistance are of high interest in the field of oncology.

Targeting of the IGF-1R promotes receptor tyrosine kinase cross-talk and leads to the subsequent compensatory activation of a myriad of downstream signaling molecules known to play a role in resistance and tumorigenic behavior(s). IR represents the closest relative to IGF-1R and is linked to anti-IGF-1R monoclonal antibody resistance via heterodimer formation (IGF-1R:IR) and/or IGF-2/IRA signaling as IRA is commonly present in a variety of solid tumors [19–21]. The fetal or A isoform of the IR appears to have a more mitogenic role in cancer cell proliferation than its purely metabolic isoform IRB [22]. The varying biological activities of IR isoforms likely relate to their differing affinities towards IGF ligands. Specifically, the metabolic IRB binds only insulin at physiologic concentrations, whereas the mitogenic IRA is able to bind and be activated by IGF-2 [23]. Thus, IRA through dimerization with IGF-1R (IGF-1R:IR) or homo-dimerization may provide mitogenic stimuli to cancer cells via IGF-2 activation. Accumulated data has implicated IRA, or the IR total content, as an important factor in breast cancer outcome and implicates IRA as a mechanism of resistance towards therapies specifically targeting IGF-1R, namely monoclonal antibody therapeutics. Patients with node negative breast cancers whose tumors express high IR, compared to even moderate IR content, report poor disease-free survival intervals [24]. Early studies have also shown that roughly 80 % of breast cancers have an IR content higher than the median content found in the normal breast and approximately 20 % of cancers present 10-fold higher levels of IR levels than the median value of normal breast tissue. Early studies targeting IGF-1R receptor in patients with refractory tumors have demonstrated that monoclonal antibody therapies may induce upregulation of insulin secretion, thereby implicating it as a compensatory mechanism that could possibly activate IR signaling as a mechanism of resistance [25].

While epithelial breast cancer cells commonly overexpress IGF-1R, surrounding tumor-associated stroma provides a rich source of IGF-2 [26]. Upon binding to IGF-2, both IR-A and IGF-1R:IR hybrid receptors are thought to promote proliferative and tumorigenic behavior predominantly via MAPK and PI3K/Akt signaling, both of which converge upon the mTOR/S6K1 axis as a critical mediator of cell growth, survival and metabolism [27]. Negative feedback inhibition of the IGF pathway via canonically active mTOR/S6K1 effectively limits IGF-1R signaling. As a result, mTOR inhibitors alleviate IGF-1R repression and increase Akt activation as a potential mechanism of resistance [3]. Moreover, a dynamic interplay between IGF and ERα in breast cancer cells has been demonstrated, where IGF-1R increase ERα phosphorylation and activity via mTOR/S6K1 and ERα mediates IGF-1R, IRS-1 and IGF ligand expression [28]. Thus, combined IGF-1R/mTOR inhibition plus hormone therapy in breast cancer cells presents as a viable therapeutic option.

## Conclusions

Data herein demonstrate that, similar to clinical trials, IGF-1R inhibition (via MK-0646) does not enhance hormonal therapy in ER-positive breast cancer cells, the results of which are may be attributable to compensatory IGF-2/IRA activity. Although IRA-induced signaling remains a potential confounder as a potential escape pathway, biochemical and *in vivo* studies support combined IGF/mTOR/ER inhibition. Importantly, for the first time, synergistic inhibition is demonstrated when combining IGF-1R and mTOR targeting agents in breast cancer cells. Finally, combined IGF/mTOR/ER inhibition was most effective in limiting tumor growth and markedly altered transcriptional activity in ER-positive breast cancer tumors.

## Additional files

Additional file 1: Table S1. Statistical analysis of *IGF1R* expression for samples depicted in Fig. 1. (XLSX 44 kb)
Additional file 2: Table S2. Statistical analysis of *IRA* expression for samples depicted in Fig. 1. (XLSX 44 kb)
Additional file 3: Table S3. Statistical analysis of *IRB* expression for samples depicted in Fig. 1. (XLSX 44 kb)
Additional file 4: Table S4. Statistical analysis of final tumor volume for samples depicted in Fig. 5. (XLSX 44 kb)
Additional file 5: Table S5. Gene expression values for all treatment cohorts expressed as relative fold-change compared to control. (XLSX 623 kb)
Additional file 6: Table S6. Ingenuity Pathway Analysis. (XLSX 115 kb)
Additional file 7: Figure S1. Pathway inhibition of the ‘Role of BRCA1 in DNA Damage Response’ in LET + MK-8669 + MK-0646 tumors. Highlighted in green are the significantly downregulated genes compared to control tumors. (PDF 415 kb)
Additional file 8: Figure S2. Heatmap depicting DNA damage gene expression across treatment cohorts. Color bar indicates log-fold expression change compared to control tumors. (PDF 92 kb)

## Abbreviations

DALO: Dalotuzumab
ERα: Estrogen receptor alpha
IGF: Insulin-like growth factor
IGF-1R: IGF-1 receptor
InsR-A: Insulin receptor alpha A
IRS: Insulin receptor substrate
LET: Letrozole
MTOR: Mammalian target of rapamycin
mTORC1: mTOR complex
RIDA: Ridaforolimus
