# Supplementary figures and images for: Ridaforolimus (MK-8669) synergizes with Dalotuzumab (MK-0646) in hormone-sensitive breast cancer
Source: BMC Cancer. 2016 Oct 20;16:814. doi: 10.1186/s12885-016-2847-3 (PMC5073873; doi:10.1186/s12885-016-2847-3)

# Supplemental Figure 1

Role of BRCA1 in DNA Damage Response

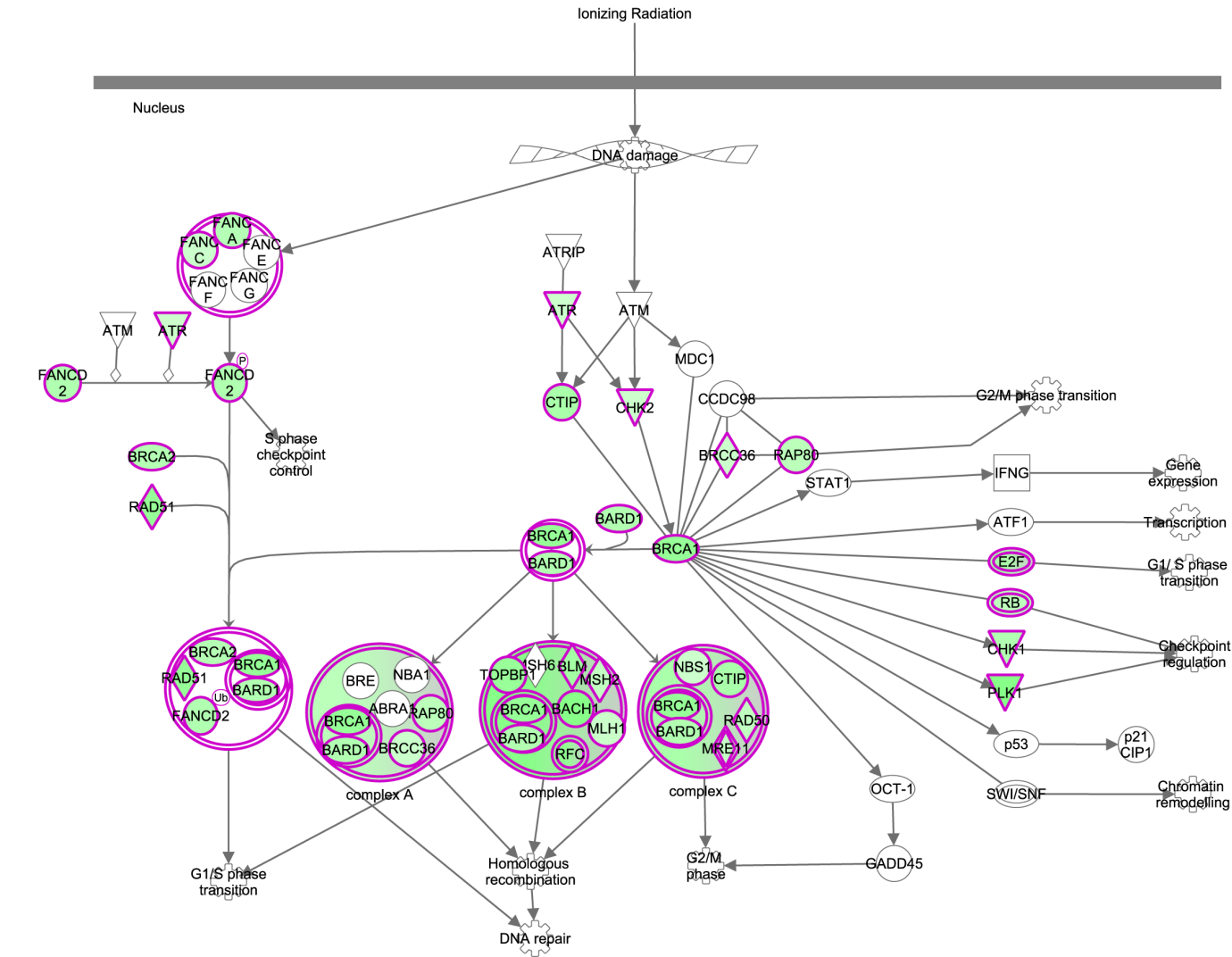

Supplement: Additional file 7: Figure S1. — Pathway inhibition of the ‘Role of BRCA1 in DNA Damage Response’ in LET + MK-8669 + MK-0646 tumors. Highlighted in green are the significantly downregulated genes compared to control tumors. (PDF 415 kb) [file 12885_2016_2847_MOESM7_ESM.pdf]

Supplemental Figure 2

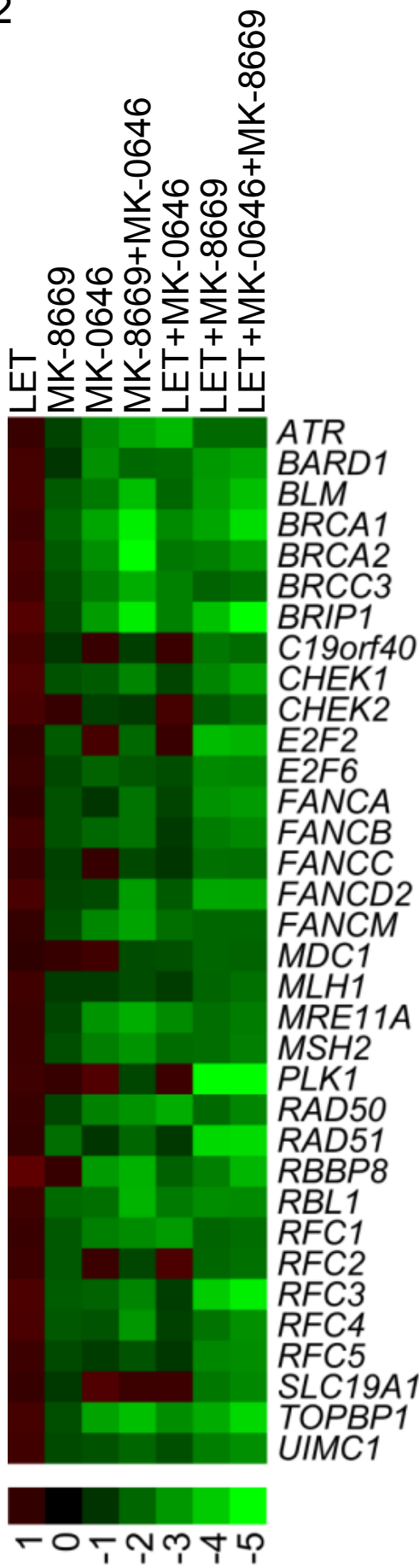

Supplement: Additional file 8: Figure S2. — Heatmap depicting DNA damage gene expression across treatment cohorts. Color bar indicates log-fold expression change compared to control tumors. (PDF 92 kb) [file 12885_2016_2847_MOESM8_ESM.pdf]
